# Autonomous molecule generation using reinforcement learning and docking to develop potential novel inhibitors

**DOI:** 10.1038/s41598-020-78537-2

**Published:** 2020-12-16

**Authors:** Woosung Jeon, Dongsup Kim

**Affiliations:** Department of Bio and Brain Engineering, Korea Advanced Institute of Science and Technology, Daejeon, Republic of Korea

**Keywords:** Virtual drug screening, Computational biology and bioinformatics, Drug discovery

## Abstract

We developed a computational method named Molecule Optimization by Reinforcement Learning and Docking (MORLD) that automatically generates and optimizes lead compounds by combining reinforcement learning and docking to develop predicted novel inhibitors. This model requires only a target protein structure and directly modifies ligand structures to obtain higher predicted binding affinity for the target protein without any other training data. Using MORLD, we were able to generate potential novel inhibitors against discoidin domain receptor 1 kinase (DDR1) in less than 2 days on a moderate computer. We also demonstrated MORLD’s ability to generate predicted novel agonists for the D_4_ dopamine receptor (D4DR) from scratch without virtual screening on an ultra large compound library. The free web server is available at http://morld.kaist.ac.kr.

## Introduction

Artificial intelligence (AI) has gained increasing interest for drug discovery^1^. One of the important goals of drug discovery using AI is to develop autonomous, de novo drug design methods^2^. Traditionally, to achieve this goal, a various combination of computational models such as quantitative structure–activity relationship (QSAR), molecular replacements, molecular simulations, and molecular docking have been used^3^. Recently, several generative models have been developed to design drug-like compounds. Many of them are based on various deep learning models such as variational autoencoder^4^, generative adversarial network^5^, and reinforcement learning^6^. One of successful examples of de novo drug design by a generative model is development of agonists of retinoid X receptors and peroxisome proliferator‐activated receptors^7^.

Remarkably, a deep generative model named GENTRL^8^ was used to develop potent inhibitors against discoidin domain receptor 1 kinase (DDR1) within 46 days, including 21 days for the molecule generation process. However, in order to ensure the success of these methods, various experimental data including activity data against drug targets of interest should be available, which is usually not the case for new drug targets. In addition, it has been pointed out that the molecules generated by GENTRL were not sufficiently different from the known active molecules in the training data set^9^. Therefore, drug design methods that do not require experimental data, especially target-specific data, are highly sought after. Another related computational approach for drug design is a virtual screening. Recently, Lyu et al. found potent agonists for the D_4_ dopamine receptor (D4DR) virtual screening on an ultra-large compound library^10^. However, virtual screening calculations on an ultra-large compound library requires technical expertise and extensive computational resources.

In this work, we introduce a new deep generative model named Molecule Optimization by Reinforcement Learning and Docking (MORLD) model for the design of potential novel inhibitors by combining reinforcement learning and docking simulation. The key feature of this approach is that the binding affinities calculated through docking simulations are given as one of rewards in the reinforcement learning. The model requires only the three-dimensional (3D) structural information of the target protein. The model does not require any training data for molecule optimization. Once provided with the target receptor structure, our model directly and automatically modifies the molecular structure of the input compound to achieve higher docking score to the target protein. Moreover, the full design process takes less than two days on a moderate computer, which is significantly shorter than the 21-day molecule generation process of GENTRL. To demonstrate the effectiveness of our method, we used our model to design potential novel DDR1 inhibitors and D4DR agonists. Evaluation of the generated compounds by various computational methods show that they have more desirable molecular properties compared to the known inhibitors.

## Results and discussion

### MORLD model

**Figure 1:**
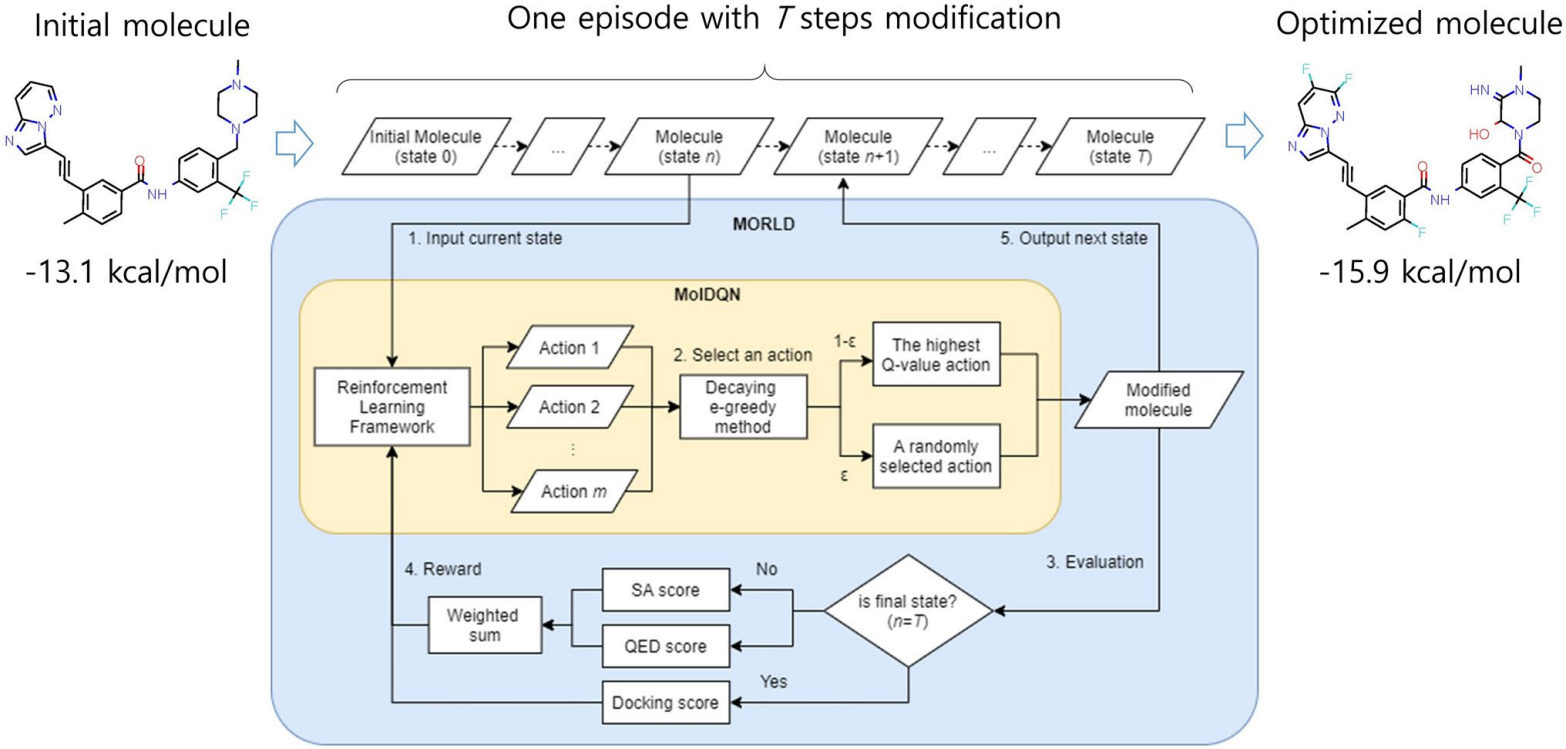
Schematic overview of the MORLD. An initial molecule is optimized by *T* steps of modifications (one episode) as shown in the flow chart. Through multiple episodes, MORLD learns a way of modifying molecules to create an optimized molecule having a higher docking score to the target protein.

A schematic overview of an optimization process in MORLD is shown in Fig. 1. In MORLD, one episode of optimization consists of *T* steps of modifications. First, the molecule of state *n* (*n* = 0 for an initial molecule) enters MORLD. Second, the input molecule is modified by MolDQN^11^. MolDQN is a framework that optimizes the properties of molecules based on the reinforcement learning and chemistry domain knowledge. We briefly summarize here how MolDQN works, but for more details, see the paper by Zhou et al. In MolDQN, a single action constitutes addition or removal of an atom or a bond in chemically valid manner, meaning that the resulting new molecule after a certain action is taken should satisfy the valence constraints, which can be checked by using software package rdkit (https://www.rdkit.org/). The atom types to be modified are specified by the user and only the bonds that satisfy the valence constraints are considered as an action. As the atom types to be considered diversify, chemical diversity of the outputs will increase, but the searching cost will also increase as well. In MolDQN, double Q-learning^12^ and bootstrapped DQN^13^ (Deep Q-Networks) is applied for reinforcement learning algorithm to learn which action (modification) brings higher rewards (achieving the given molecular properties). Among all possible actions, one action is selected for generating the next state of molecule. In this study, we used the decaying *ϵ*-greedy method in which either an action of MolDQN is selected randomly with the probability of *ϵ*, or the best action according to the current *Q* function is chosen with the probability of 1-*ϵ*. And the epsilon value gradually decreases over the episodes, from 1 to 0. Third, the modified molecule is evaluated by scoring functions. Here, different scoring functions are applied depending on the state. If the state is not the terminal state (*n* < *T*), the modified molecule is evaluated by the synthetic accessibility (SA) and the quantitative estimate of drug-likeness (QED) scores. SA score was developed to estimate the ease of synthesis of drug-like molecules^14^. QED score is a quantitative estimate for how similar particular molecules are to the known drugs in terms of various physicochemical properties and structural features^15^. These scores encourage MORLD to generate compounds that are easy to synthesize and have physicochemical properties and structural features similar to those of the known drugs. Fourth, the weighted sum of two scores is given as a reward of MolDQN. Fifth, the modified molecule becomes as the molecule of the next state. The above process is repeated until it reaches the final state, state *T*. When the state is final, the modified molecule is docked by QuickVina 2^16^, which is known to be slightly less accurate but faster than Autodock Vina^17^, against the target protein. The docking score of QuickVina 2 is given as a reward of MolDQN. Then the molecule at the final state becomes the result of optimization and one episode of optimization ends. Therefore, in each episode, one molecule is generated. As a consequence, there are the same number of generated molecules as the number of episodes. MORLD repeats the episodes for a given number of times. At the same time, MolDQN tries to reduce the gap between the expected future reward of the actions (known as *Q*-value) and the real rewards of the chosen action from the experiences. Through the experiences of many episodes, the expected future rewards gradually approximates the actual reward values. In other words, it learns which action brings higher rewards in the future. Eventually, after sufficient number of episodes, MORLD steadily generates potential novel inhibitors with higher docking score (along with high SA and QED scores) to the given protein structure.

### Validation of MORLD

To assess the validity of MORLD, we built a control model (random model) that modifies the structure of compounds by randomly selected actions contrary to the *Q*-value-based action of MORLD. We compared the optimization results of the random model with those of MORLD for the protein target DDR1. We used the protein structure of DDR1 (PDB ID: 3ZOS) as a target protein structure. The initial lead molecule for both models was “ponatinib”, the ligand of 3ZOS, and the binding site information was also derived from 3ZOS.

**Figure 2:**
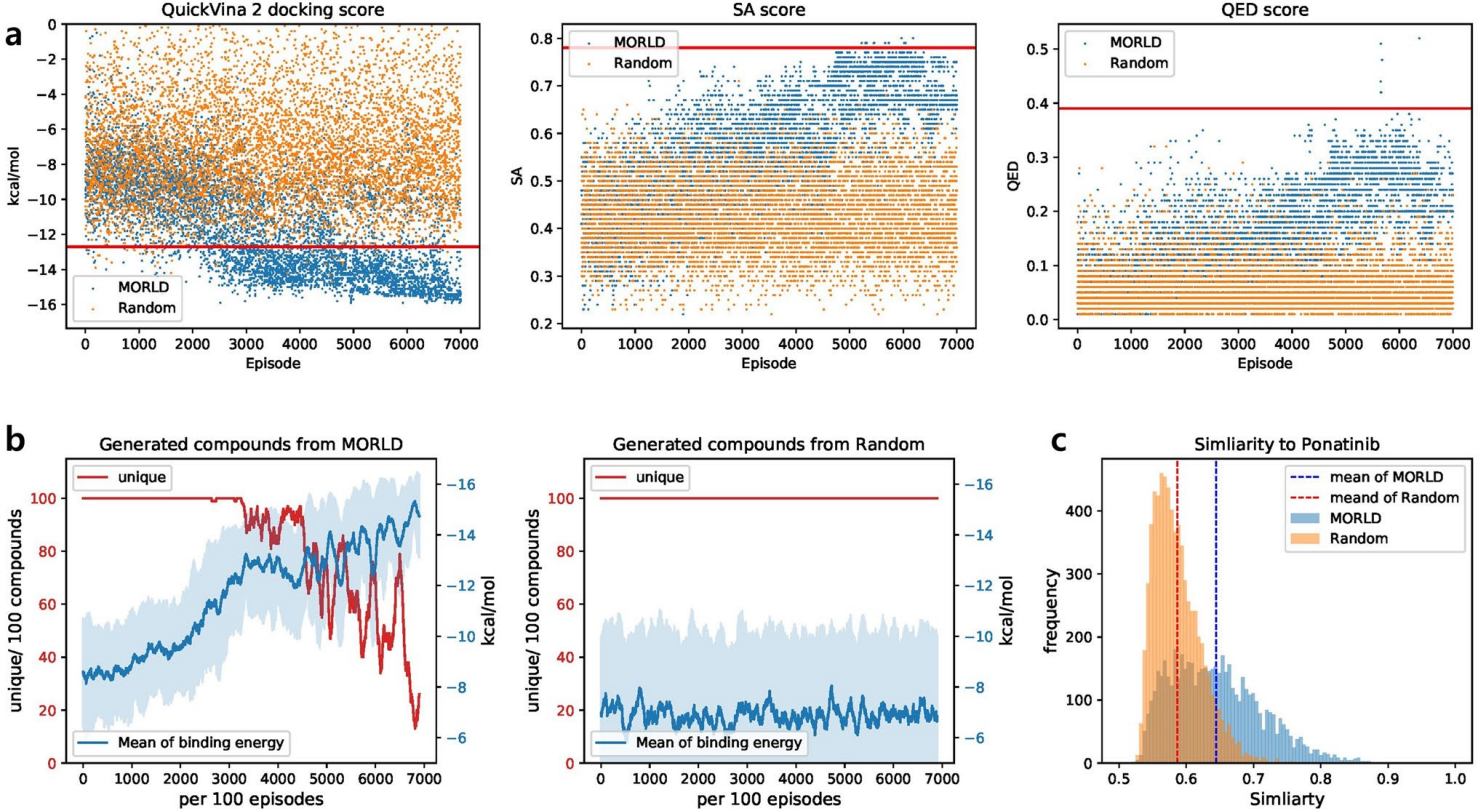
Comparison of the compounds from MORLD and from random model. (**a**) Molecular properties (QuickVina 2 docking score, SA score, and QED score) of the compounds generated by MORLD and the random model. The Red horizontal line indicates the molecular property of the initial molecule. (**b**) The number of unique compounds generated for each 100 episodes (red solid line) and the mean of QuickVina 2 docking scores (blue solid line) of the generated compounds from MORLD (left) and random model (right). The standard deviation of QuickVina 2 docking scores is depicted as blue area. (**c**) Tanimoto score of the generated compounds from MORLD (blue area) and random model (orange area) against the lead compound (“ponatinib”).

Figure 2a shows the molecular properties (QuickVina 2 docking score, SA score, and QED score) of generated compounds from MORLD (blue) and the random model (orange). The scores were measured at the end of each episode. The molecular properties of the initial molecule (ponatinib) are marked as red solid line. MORLD clearly improved the molecular properties of generated compounds as the training proceeded. At first, the generated compounds were not distinguishable from those from the random model. It is because MORLD uses decaying *ϵ*-greedy method for exploration. In the early episodes of MORLD, near 0-th episode, MORLD takes random action with the high probability of *ϵ* close to 1 rather than greedy action. Therefore, it is likely to score bad in early episodes in MORLD. However, MORLD gradually reduced the probability of taking random actions and increased the probability of taking greedy action as the episode proceeded, and as the training proceeds, MORLD starts to learn which action brings higher rewards. After enough training time, MORLD was able to steadily generate the molecules with better docking score while the random model could not. SA and QED scores of the generated compounds from MORLD were also noticeably higher than those from random model, and getting closer to the scores of the lead compound. The SA and QED scores of the early episodes in MORLD and the random model show much lower values than its initial molecule. Usually, molecules with high SA and QED scores have specific substructures and patterns that are found in the existing drug-like molecules. For the molecules generated from the early episodes in MORLD or the random model, it was difficult to get such substructures. However, unlike the random model, MORLD was able to learn the patterns of molecules with high SA and QED scores through the training. We conclude that MORLD with decaying *ϵ*-greedy method is much more efficient than the random selection, and MORLD can optimize the multiple molecular properties with sufficient training.

Next, we investigated whether MORLD could generate non-redundant compounds as the training proceeded (Fig. 2b). To do this, we counted the number of unique compounds for each 100 episodes and calculated the mean docking scores of those compounds. Figure 2b shows that MORLD could generate the compounds with better docking score as the training proceeded; the average docking score of MORLD compounds was nearly − 16 kcal/mol at the end of training, ~  − 3 kcal/mol smaller than the docking score of its lead compound. In contrast, the mean of docking score of the compounds from random model did not improve. However, the number of unique compounds from MORLD began to decrease after around 3000th episode. Near the 7000th episode, about 80% of generated compounds were redundant. As already mentioned in the original MolDQN paper^11^, the reason is that the reinforcement learning algorithm in MORLD tends to follow the single optimal policy to increase the given reward. Therefore, after the enough training, MORLD only acts according to the optimal policy learned from training, resulting in a limited number of non-redundant compounds.

Lastly, we checked the diversity of generated compounds, as well as the similarity to its lead compound (Fig. 2c). Here we removed the redundant compounds in each generated compound set and calculated Tanimoto score of the compounds against the lead compound based on the extended-connectivity fingerprint (ECFP) of the compounds^18^. The result shows that the compounds from MORLD were significantly similar to the lead compound than the compounds from the random model (Wilcoxon rank-sum test p-value < 1e − 10). The mean value of similarity of MORLD compounds and random compounds are 0.644 and 0.587, respectively. Nevertheless, it is clear from Fig. 2c that MORLD could generate sufficiently diverse compounds whose Tanimoto score against the lead compound broadly ranged from 0.5 to 0.8.

### Design of DDR1 inhibitors

**Figure 3:**
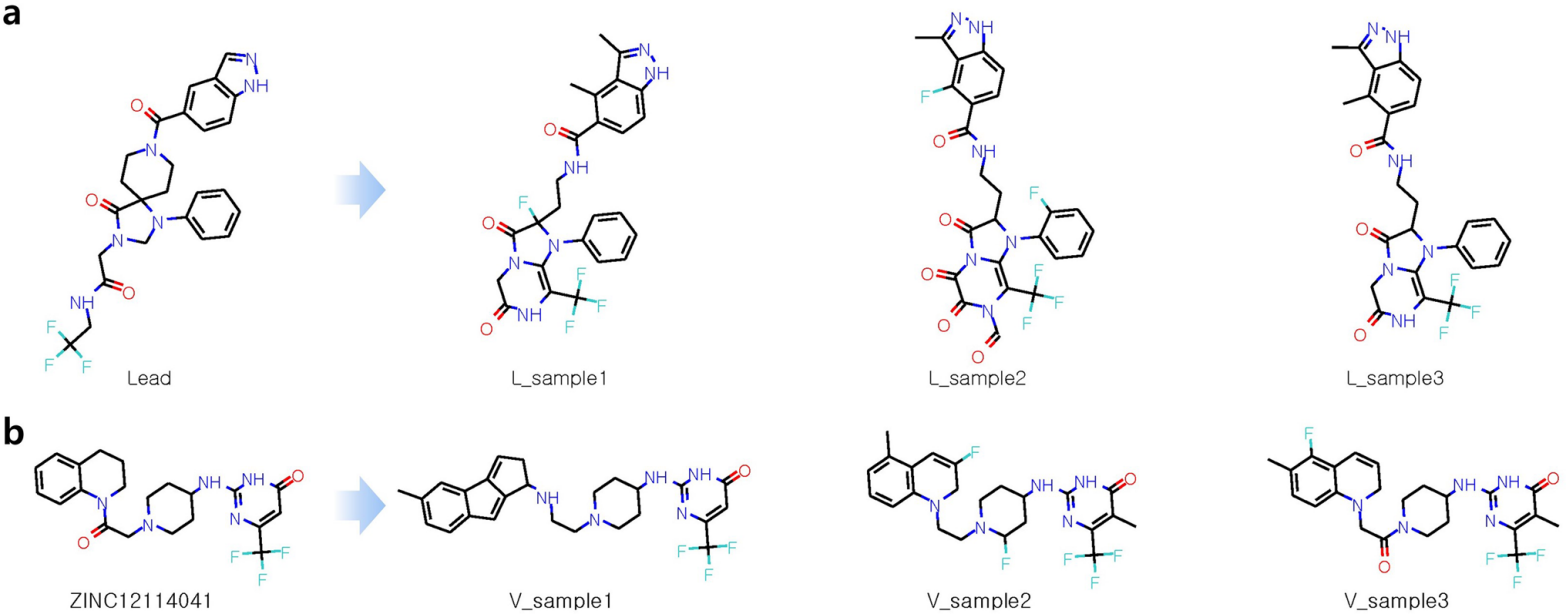
Samples from the inhibitors generated by the MORLD for the target DDR1. (**a**) Using the “Parent structure” shown in Fig. 1 of Zhavoronkov’s paper as the initial lead compound (Lead), DDR1 inhibitors were generated and three sample compounds are shown. (**b**) Three sample compounds generated from the initial compound ZINC12115041, which was identified by a simple virtual screening procedure for DDR1.

We first applied MORLD to generate predicted novel inhibitors against DDR1. In a recent publication^8^, Zhavoronkov et al. demonstrated that it was possible to discover potent DDR1 kinase inhibitors within 46 days, including 21 days for the model training and molecule generation steps. We used the protein structure of DDR1 (PDB ID: 3ZOS) as a target protein structure. Several samples generated by MORLD and their docking scores along with SA and QED scores are shown in Fig. 3 and Table 1. To evaluate docking scores of the generated molecules from MORLD, we used AutoDock Vina which is known to be slightly more accurate than QuickVina 2. In addition to AutoDock Vina, docking scores were also calculated by two other popular docking programs (rDock^19^ and Ledock^20^) for evaluation, because reliability of the scoring functions of current docking methods are not sufficiently high and one way to alleviate this problem is to use consensus scoring of multiple docking methods^21^. First, we assumed that a lead compound is available for optimization. We selected the “Parent structure” shown in Fig. 1 of Zhavoronkov’s paper as a lead compound (Lead) and generated new optimized compounds. Those compounds are listed in Supplementary Table S1, and several samples are shown in Fig. 3a. In another situation, we assumed that no lead compound is available. For this situation, we first identified ZINC12114041 by performing a small-scale virtual screening against DDR1 using MTiOpenScreen^22^, and subsequently used MORLD to generate new predicted inhibitors. Several samples generated through this process are shown in Fig. 3b.

**Table 1:** Docking scores calculated by four popular docking programs (AutoDock Vina, QuickVina 2, rDock, Ledock), as well as SA and QED scores for the DDR1 inhibitors.

Compounds	Vina* (QuickVina 2)	rDock*	Ledock*	SA**	QED***
Lead (initial)	− 7.6 (− 7.0)	− 21.24	− 6.50	0.75	0.55
L_Sample1	− 12.4 (− 12.6)	− 40.27	− 10.06	0.68	0.35
L_Sample2	− 12.5 (− 12.6)	− 40.91	− 10.31	0.67	0.2
L_Sample3	− 12.4 (− 12.5)	− 39.55	− 10.10	0.7	0.49
ZINC12114041 (initial)	− 10.9 (− 11.0)	− 30.31	− 6.90	0.81	0.77
V_Sample1	− 13.1 (− 13.1)	− 36.27	− 8.69	0.69	0.6
V_Sample2	− 13.1 (− 13.1)	− 36.68	− 8.27	0.63	0.49
V_Sample3	− 13.1 (− 13.1)	− 37.09	− 8.23	0.75	0.66
Ponatinib (initial)	− 12.7 (− 12.7)	− 43.02	− 11.79	0.78	0.39
P_Sample1	− 15.9 (− 15.9)	− 50.08	− 13.59	0.65	0.2
Compounds 1 (active)	* − 13.0 (− 13.0)*	* − 42.13*	* − 11.93*	*0.76*	*0.38*
Compounds 3 (moderate)	* − 11.7 (− 11.3)*	* − 34.95*	* − 7.68*	*0.71*	*0.84*
Compounds 5 (inactive)	* − 9.3 (− 10.4)*	* − 25.96*	* − 7.46*	*0.76*	*0.54*

Data in italics indicates compounds generated by Zhavoronkov et al.^8^.

*For docking scores, the lower, the better. The unit of Vina, QuickVina 2, and Ledock score is kcal/mol.

**Synthetic accessibility.

***Quantitative Estimate of Drug-likeness.

We compared the docking scores and other properties of our generated compounds to those of the compounds (Compounds 1, 3, and 5) generated by Zhavoronkov et al. The results shown in Table 1 indicate that all the generated compounds have docking scores that are better than or comparable to those of the experimentally validated active compounds (Compounds 1 and 3). Moreover, the observation that Compound 1 (active) has a better docking score than Compound 3 (moderate), which in turn has a better score than Compound 5 (inactive) gives us some confidence that the docking calculations performed reasonably well for DDR1. Taken together, these findings strongly suggest that it is possible to generate predicted novel inhibitors against DDR1 using MORLD without any training data, aside from the target structure, in less than 2 days.

### Design of D4 dopamine receptor

**Figure 4:**
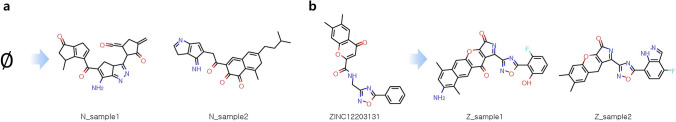
Samples from the inhibitors generated by the MORLD for the target D4DR. (**a**) Sample D4DR agonists generated from scratch (None). (**b**) Sample D4DR agonists generated from ZINC12203131, which was found by virtual screening.

In a recent publication^10^, Lyu et al. reported that they were able to develop potent D4DR agonists by virtual screening on an ultra-large compound library. In order to evaluate how effective MORLD method is compared to virtual screening on an ultra-large compound library, we applied MORLD to design agonists for D4DR. The target structure and binding site information was taken from PDB ID: 5WIU, the same structure used by Lyu et al. Several sample compounds and their molecular properties are shown in Fig. 4 and Table 2. For this target, we assumed that lead compound information was not available. First, we examined whether MORLD could generate agonists without using initial compound structure. Remarkably, we were able to successfully generate potent agonists (N_sample1, N_sample2) from scratch (None) without providing any initial lead compound information, suggesting that MORLD can replace and outperform virtual screening calculations on an ultra-large compound library which requires extensive expert knowledge and computational resources. Next, we first identified an initial lead compound (ZINC12203131) and then used MORLD to generate optimized compounds (Z_sample1, Z_sample2). Molecular properties of generated compounds compared to those of experimentally verified active compounds (ZINC465129598, ZINC518842964, ZINC464771011) shown in Table 2 strongly suggest that MORLD could generate potent D4DR agonists, even from scratch.

**Table 2:** Docking scores calculated by four popular docking programs (AutoDock Vina, QuickVina 2, rDock, Ledock), as well as SA and QED scores for the D4DR agonists.

Compounds	Vina* (QuickVina 2)	rDock*	Ledock*	SA**	QED***
None (initial)	–	–	–	–	–
N_Sample1	− 12.7 (− 12.7)	− 39.80	− 7.51	0.5	0.57
N_Sample2	− 11.2 (− 11.3)	− 37.99	− 7.66	0.61	0.41
ZINC12203131 (initial)	− 10.9 (− 10.8)	− 36.41	− 6.99	0.85	0.59
Z_Sample1	− 14.3 (− 14.3)	− 40.82	− 8.46	0.75	0.69
Z_Sample2	− 13.8 (− 13.8)	− 39.38	− 8.14	0.76	0.67
ZINC465129598 (active)	* − 10.1 (− 9.9)*	* − 35.61*	* − 7.33*	*0.74*	*0.76*
ZINC518842964 (active)	* − 9.3 (− 9.3)*	* − 34.88*	* − 6.65*	*0.79*	*0.91*
ZINC464771011 (active)	* − 8.3 (− 8.2)*	* − 29.86*	* − 5.91*	*0.76*	*0.86*

Data in italics indicates active inhibitors from Lyu et al.^10^.

*For docking scores, the lower, the better. The unit of Vina, QuickVina 2, and Ledock score is kcal/mol.

**Synthetic accessibility.

***Quantitative estimate of drug-likeness.

### Analysis of docking poses

**Figure 5:**
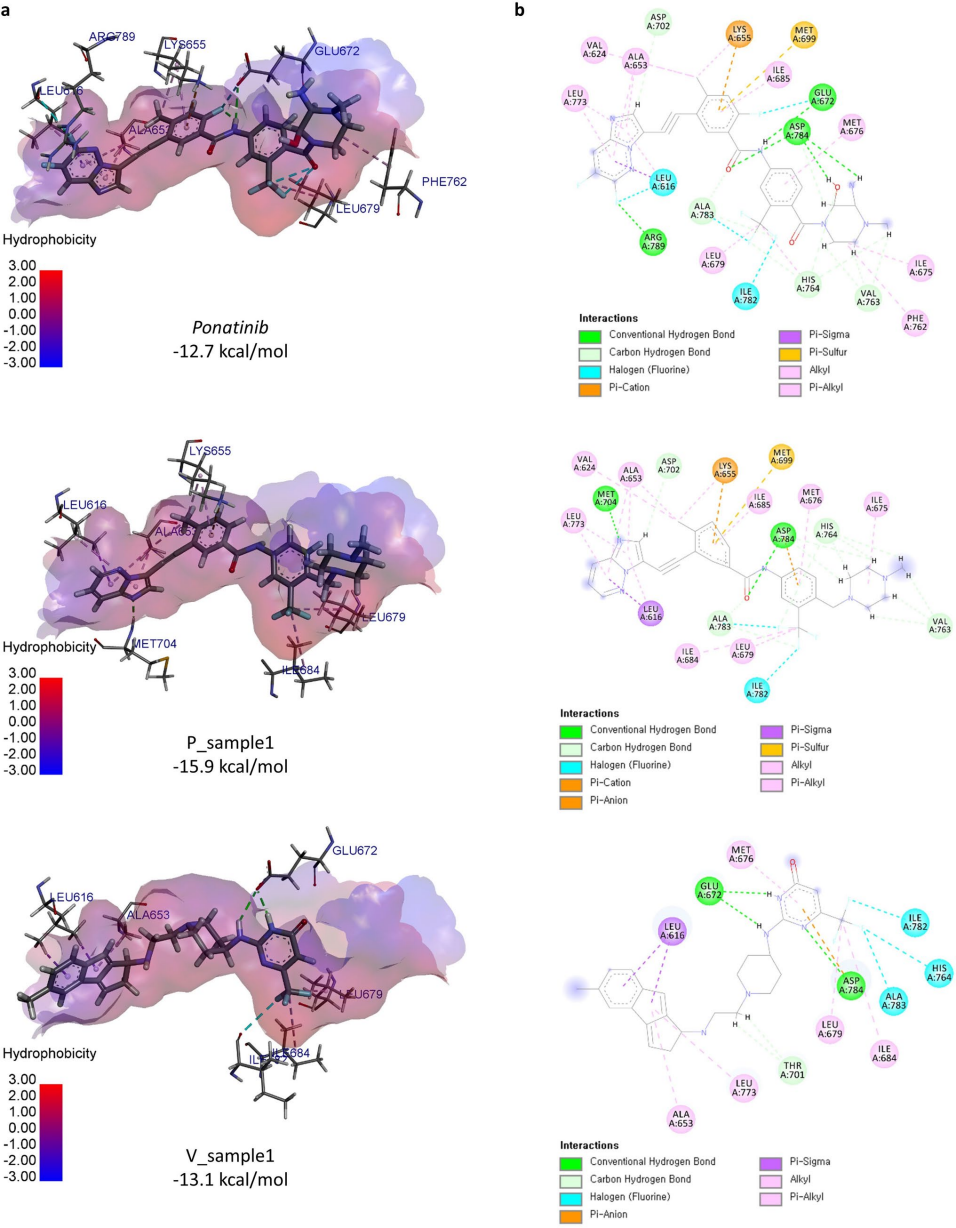
Docking poses of ponatinib, P_sample1, and V_sample1. (**a**) The X-ray crystallographic pose of ponatinib from PDB ID:3ZOS (native), P_sample1 (optimized from ponatinib) docked into 3zos, and V_sample1 (optimized from ZINC12114041) docked into 3zos. (**b**) Binding interactions of ponatinib, P_sample1, and V_sample1 to 3ZOS.

To understand why docking scores of optimized molecules increase, we analyzed the docking poses and interactions of generated molecules. We compared the docking pose of P_sample1, and V_sample1 (Fig. 5a). Ponatinib is a baseline that has a crystallographic binding structure with DDR1, PDB ID: 3ZOS. P_sample1 is one of the sample molecules optimized from ponatinib using MORLD against the target 3ZOS and also incorporated in the result of “Validation of MORLD” part. P_sample1 is relatively an easier case for lead optimization since it was optimized from ponatinib and 3ZOS has the native structure of binding pocket for ponatinib. On the other hand, V_sample1 was optimized from ZINC12114041 that was obtained from virtual screening, which is not the native ligand of 3ZOS, therefore it is more challenging compared to the optimization from ponatinib. However, V_sample1 is a more realistic case for designing novel drugs, i.e., virtual screening followed by lead optimization.

As expected, the docking pose of P_sample1 is highly similar to that of ponatinib, while the docking pose of V_sample1 is partially aligned with that of ponatinib (Fig. 5b). To visualize the binding interactions, we used BIOVIA Discovery Studio Visualizer^23^. When comparing the binding poses of ponatinib and P_sample1, we notice that many residues such as A653, K655, and L679 participate in the same types of interactions in both molecules, while some of residues (L616, and D784, etc.) maintain the interactions in both molecules but of different types. The interaction with M704 and I684 is missing in P_sample1 but there are also newly created interactions such as interactions with E672, F762 and R789. We observe that newly created structures are highly involved in interactions. Compared to the lead molecule, ZINC12114041, V_sample1 has new interactions with I684, I701, and I782 (Supplementary Fig. S1). Such interaction changes are likely to increase the docking score of V_sample1 compared to ZINC12114041 (− 10.9 kcal/mol).

### Comparison with other methods

GENTRL, used as a benchmark method in this study, is a generative model for designing novel inhibitors. Both MORLD and GENTRL are based on reinforcement learning, but, unlike GENTRL, MORLD does not require any training data for molecule optimization except for the 3D structure of a target protein. GANDI^24^, MoleGear^25^, Ligbuilder3, and AutoGrow4^26^ optimize or generate molecules based on genetic algorithm. These methods employ fragment-based approach that modifies molecules by joining or replacing small fragments from existing molecules. Notably, Autogrow4 utilizes the pre-defined reactions when combining fragments. Another fragment-based approach is de novo DOCK^27^, which generates molecules by linking fragments by DOCK anchor‐and‐grow strategy^28^. In contrast, MORLD is an atom-based approach that adds or removes only one atom or bond at each step of modifications. Compared to all these methods, MORLD have several advantages. MORLD requires relatively small amount of computational resources. Moreover, MORLD has an easy-to-use free web server, which makes downloading and installing software packages unnecessary. In addition, as in some other open source programs, the source code is open to public so that a user can download and modify the code, e.g., add or modify criteria for rewards, for their own purpose, such as replacing docking software from QuickVina 2 to Autodock Vina or some other program.

For performance comparison, as a benchmark test, we compared the optimization results from MORLD to those from AutoGrow4, which was published most recently and the most similar method to ours. Here we tried to compare the results of molecule optimization starting from the same lead. We believe that this would make it fairer to compare the optimization results. We chose “Compound 3”, one of the high scoring compound from Spiegel et al.^26^ (AutoGrow4) with known lead information, for the comparison of the optimization performance. We optimized E7449, the same lead as “Compound 3” shown in Fig. 2 of the paper by Spiegel et al., to generate predicted inhibitors against PARP-1 catalytic domain using MORLD. The same protein structure (PDB ID: 4R6E) was used for optimization. The coordinate of binding site and grid box size were the same as provided in the work of Autogrow4. Here, the docking scores of generated molecules from MORLD were measured by QuickVina 2 as in Autogrow4. The optimized results from MORLD are described in Supplementary Table S1.

A_sample1 (Supplementary Fig. S2), one of the arbitrarily picked sample among the generated molecules using MORLD, shows docking score of − 14.6 kcal/mol which is better than its lead, E7449 fragment (− 8.6 kcal/mol). On the other hand, “Compound 3” from Spiegel et al., which was also optimized from E7449 fragment, has the docking score of − 12.2 kcal/mol when the same docking scheme used in MORLD was also employed (in Spiegel et al., it was reported to be − 14.8 kcal/mol). Moreover, SA and QED scores of A_sample1 (0.65 and 0.56 respectively) are better than those of Compound 3 (0.54 and 0.51). Visual inspection reveals that A_sample1 conserves the π–π stacking interaction with Y907 and Y896 and the hydrogen bond with G863 that are well known binding interactions^29–36^. In addition, the interacting residues H862 and Y896 are well conserved residues from PARP-1 to PARP-6^37^. These results indicate that MORLD preserves the important interactions with PARP-1 while increasing the docking score.

## Conclusion

In this work, we developed an autonomous molecule generation method named MORLD that automatically generates and optimizes lead compounds by combining reinforcement learning and docking. This model requires only a target protein structure and directly modifies ligand structures to obtain higher predicted binding affinities, docking scores, for the target protein. For a given target protein structure, using our model, one can generate molecules with high predicted binding affinity to the target protein without relying on any target-specific training data. We demonstrated that the MORLD model was highly efficient compared to the random searching strategy model. Moreover, we demonstrated that for the two targets, DDR1 and D4DR, MORLD could successfully generate optimized molecules whose molecular properties were better than or comparable to those of experimentally examined compounds. We also demonstrated that MORLD could be successfully employed for three different situations; (i) when an active lead compound is available, (ii) when an initial lead compound can be identified by virtual screening procedure, and (iii) when starting from scratch.

There are several limitations in our model that need to be addressed. First, due to the inherent limitations of docking simulations^38^, better docking scores do not guarantee higher binding affinities. In addition, MORLD may not be applicable when the 3D structure of targets is not available, e.g., intrinsically disordered proteins and the proteins without druggable binding pockets. Second, the Q-values of MORLD are calculated from the extended-connectivity fingerprint (ECFP) representation of the compounds^18^. However, ECFP may not properly represent 3D structural information of the ligands. Third, because the reinforcement learning algorithm of MORLD is trained to select the action with the highest Q-value, the model tends to design a limited number of optimized compounds. Finally, because MORLD is an atom-based model, it is difficult to explore all the chemically valid space due to its combinatorial nature. As a result, when a limited number of episodes are used for training the model, the many different suboptimal outcomes can be obtained depending on the initial search direction, especially in the de novo design. Accordingly, some trials may generate molecules having more appropriate structures than other trials. In addition, because there is no other requirement except from chemical validity when adding or removing an atom or a bond, and the SA and QED scores are not perfect, the model may generate molecules with chemically inadequate substructures.

Despite these limitations, MORLD has many attractive features that make it a unique and highly valuable tool. First, MORLD does not require any training data except the target protein structure, which makes MORLD an ideal tool for novel drug targets. Second, MORLD does not require a model building and training procedure, which makes it possible for anyone without modeling expertise to use MORLD. Third, MORLD can be used as a tool complementary to a virtual screening procedure on an ultra-large compound library which can be computationally expensive. Finally, there is a public server (http://morld.kaist.ac.kr) that is easy to use and runs relatively fast which can allow drug developers to obtain immediate results for their target proteins.

## Methods

### Docking simulation in MORLD

For docking in MORLD model, we implemented QuickVina 2 (version 1.1.2)^16^ which improves the docking speed over AutoDock Vina without sacrificing docking accuracy. Faster docking helps to reduce the training time of MORLD model. QuickVina 2 requires three information for docking: (1) the 3D structure of a ligand compound, (2) the 3D structure of the target protein, and (3) the binding site information. We used “open babel^39^” to generate the 3D conformation of the ligand from the SMILES format. The ligands are protonated using open babel appropriate at pH 7. The 3D structure of the target protein and binding site information are derived from Protein Data Bank. We protonated the target proteins using PDB2PQR^40^ server (version 2.1.1) with default setting and pH 7 and PQR file, output of PDB2PQR, is converted to PDB by open babel. QuickVina 2 calculates the predicted binding energy between the target protein and the ligand within the given search space at the binding site. The binding sites are derived from the native ligands in the PDB structures.

### Reward design

We used three different scores to define the reward of MORLD model: SA score, QED score, and docking score. SA (Synthetic Accessibility) score measures the ease of synthesis of compounds based on its substructures. The SA score ranges from 1 (easy to make) to 9 (hard). We used a simple normalized SA score from You et al.^41^.

$$Normalized \,{Score}_{SA}= \frac{10-{Score}_{SA}}{9}$$

In short, here, we call the normalized SA score “SA score”. Molecules having high SA score tend to have substructures that are frequently found in existing molecules, but merely having high SA scores does not guarantee that they are easily synthesizable.

QED (Quantitative Estimate of Drug-likeness) score measures how similar a molecule is to a drug. The QED score ranges from 0 (less drug-like) to 1 (more drug-like). The QED score as a reward keeps the drug-likeness property of the optimized compounds.

Docking score in the MORLD model is given as a negative value of the binding energy calculated by QuickVina 2 as a reward, since the lower binding energy means the more stable state and the stronger predicted binding affinity. To reduce the training time of the model, we exploited the docking score only at the terminal state rather than during executing the docking at all steps. Before the terminal state, we only used weighted sum of SA and QED scores. The reward of state $s$ at time step $t$ (${r}_{s,t}$) is defined as,

$$r\left(s, t\right)= \left\{\begin{array}{l}\left({w}_{SA}*{Score}_{SA}\left(s\right)+{w}_{QED}*{Score}_{QED}\left(s\right)\right)*{\upgamma }^{T-t}, t<T\\ -{Score}_{Docking}\left(s\right), t=T\end{array}\right.,$$

where ${Score}_{S{A}_{norm}}(s)$, ${Score}_{QED}(s)$, and ${Score}_{Docking}(s)$ correspond to SA score, QED score, and docking score from QuickVina 2 of state $s$, respectively. *T* is the maximum number of steps in one episode. ${w}_{SA}$ and ${w}_{QED}$ are weight values for SA and QED score respectively. If ${w}_{SA}$ is 0, MORLD will not consider SA score and the same for ${w}_{QED}$. If the weight values are large enough, e.g. larger than 1, MORLD relatively more focuses on SA and QED score than the docking score. In this study, ${w}_{SA}$ and ${w}_{QED}$ are set to 1 but these values can be changeable according to the user’s purposes. We added ${\upgamma }^{T-t}$ term to weigh more the rewards that are closer to the terminal step.

### Experimental design

Using MORLD model, we generated predicted novel inhibitors against two target proteins: discoidin domain receptor 1 (DDR1) and D_4_ dopamine receptor (D4DR). We compared the docking scores of the optimized compounds to those of the experimentally verified inhibitors of the two target proteins. Here we used three different docking methods for cross-checking: (1) AutoDock Vina (version 1.1.2), (2) rDock (version 2013.1), and (3) Ledock (version 1.0). We also compared SA and QED scores of generated compounds with those of experimentally verified inhibitors.

To generate the potential novel inhibitors of DDR1, we used two lead compounds: (1) “parent compound” (Lead), and (2) ZINC12114041. The “parent compound” is described as the parent structure of “Compound 1, 3, and 5” from Zhavoronkov et al., and ZINC12114041 is the potential inhibitor identified by a simple virtual screening method, MTiOpenScreen^22^, against DDR1. In virtual screening, the target protein structure was PDB ID: 3ZOS and binding site information was taken from the ligand binding site of 3ZOS. The result of virtual screening against DDR1 is described in Supplementary Table S2. The coordinate of the binding site was set to (− 7.5, 2.5, − 40) along x, y, and z-axis, respectively, and the size of the search space was set to (24, 20, 20) Å.

For the benchmark dataset of DDR1 inhibitors, we took three compounds, “Compound 1, 3, and 5” from Zhavoronkov et al. “Compound 1” is strong inhibitor against DDR1 (${IC}_{50}(DDR1)$=10 nM), “Compound 3” a moderate inhibitor (${IC}_{50}(DDR1)$=1000 nM), and “Compound 5” an inactive (${IC}_{50}\left(DDR1\right)>{10}^{4}$ nM). The docking scores of the compounds from Zhavoronkov et al. were calculated with three docking methods and 3ZOS, the same structure as Zhavoronkov’s paper.

We generated the potential novel D4DR agonists in two different ways: (1) without the lead (None) and (2) using the lead, ZINC12203131. In the first approach, we generated the predicted inhibitors from scratch without using any other experimental data. It is more challenging task than generating predicted inhibitors using initial leads. The second approach used the lead, ZINC12203131, which was brought from the virtual screening, MTiOpenScreen. The result of virtual screening against D4DR is described in Supplementary Table S2. For virtual screening and optimization, the target structure and binding site information was taken from PDB ID: 5WIU which is the same structure as used in Canon et al. The coordinate of the binding site is set to (− 17, 15, − 18) along x, y, and z-axis, respectively, and the size of the search space was set to (24, 12, 24) Å.

We took three active inhibitors from Lyu et al.^10^ for the benchmark dataset of D4DR agonists: (1) ZINC465129598 (${K}_{i}^{{D}_{4}}=80\, \text{nM}$), (2) ZINC518842964 (${K}_{i}^{{D}_{4}}=120\, \text{nM}$), and (3) ZINC464771011 (${K}_{i}^{{D}_{4}}=140\, \text{nM}$). The docking scores of three compounds were calculated against D4DR structure (PDB ID: 5WIU) as well.

### Sampling method

To select the best compounds among many candidates, various drug properties including docking score should be considered. In addition the docking score, we considered SA and QED scores as well. We first sorted the molecules by Quickvina 2 docking score, and then selected the molecules for which all three scores were reasonably high and the structures were visually acceptable. At MORLD web server, we provide all three scores of generated molecules in the result file, so users can choose molecules having high scores for their preferences. Because SA and QED scores are not perfect measures, we recommend users to check the structures of the generated molecules visually whether those structures are chemically reasonable.

### Hyperparameters

Table 3 shows the hyperparameter setting for each experiment. The number of step is the maximum number of modification actions in one episode. And only the given atom types are added. The other hyperparameters of MolDQN used in MORLD are described in Supplementary Data 1.

**Table 3:** Hyperparameters in MORLD.

Initial molecule	Target structure	Num. of steps	Num. of episodes	Atom types	Weight of SA	Weight of QED
Ponatinib	3ZOS	20	7000	C, N, O, F	1	1
Lead	3ZOS	20	7000	C, N, O, F	1	1
ZINC12114041	3ZOS	20	7000	C, N, O, F	1	1
None	5WIU	48	15,000	C, N, O	1	1
ZINC12203131	5WIU	20	7000	C, N, O, F	1	1
E7449 frag	4R6E	24	20,000	C, N, O, F	1	1

### Equipment

We used one GPU with Nvidia GTX2080Ti and one CPU with Intel Xeon Silver 4114 CPU @ 2.20 GHz with 20 cores for training RL model and ran docking simulation. MORLD web server also has the same hardware specification and additionally a CPU node with 24 cores of INTEL XEON SILVER 4214 CPU @ 2.20 GHz is supported. Although the experimental time is depends on hyperparameters, in the case of ponatinib, it took lesser than 2 days to finish one session of optimization.

## Supplementary Information

Supplementary Information.

Supplementary Figure S1.

Supplementary Figure S2.

## Data Availability

Raw generation results and virtual screening results are available from the authors upon request.

## Code availability

Code is available at http://github.com/wsjeon92/morld.
