# Supplementary material for: Autonomous molecule generation using reinforcement learning and docking to develop potential novel inhibitors
Source: Sci Rep. 2020 Dec 16;10:22104. doi: 10.1038/s41598-020-78537-2 (PMC7744578; doi:10.1038/s41598-020-78537-2)
Supplement: Supplementary file 1 — Supplementary Information. [file 41598_2020_78537_MOESM1_ESM.docx]

Supplementary Information:

Autonomous Molecule Generation Using Reinforcement Learning and Docking to Develop

Potential Novel Inhibitors

Woosung Jeon, Dongsup Kim*

Department of Bio and Brain Engineering, Korea Advanced Institute of Science and Technology, Daejeon,

Republic of Korea

*Correspondence to [kds@kaist.ac.kr](mailto:kds@kaist.ac.kr)

Supplementary Table 1. Top 10 predicted inhibitors with the highest QuickVina 2 (Qvina) docking score optimized by MORLD.

| Target  (PDB ID) | Initial molecule | SMILES | Qvina score | SA | QED | Remarks |
| --- | --- | --- | --- | --- | --- | --- |
| DDR1  (3ZOS) | *ponatinib* | C=C(c1ccc(NC(=O)c2ccc(C)c(C#Cc3c(O)nc4cc(F)c(F)nn34)c2)cc1C(F)(F)F)N1C(=C)C2NC(=O)CN2CC1=O | -15.9 | 0.61 | 0.22 |  |
| DDR1  (3ZOS) | *ponatinib* | Cc1c(C#Cc2c(O)nc3cc(F)c(F)nn23)cc(C(=O)Nc2ccc(C(=O)N3C#CN(C)CC3)c(C(F)(F)F)c2)c(F)c1F | -15.9 | 0.64 | 0.26 |  |
| DDR1  (3ZOS) | *ponatinib* | Cc1cc(F)c(C(=O)Nc2ccc(C(=O)N3CCN(C)C(=N)C3O)c(C(F)(F)F)c2)cc1C#Cc1c(O)nc2ccc(F)nn12 | -15.9 | 0.65 | 0.2 |  |
| DDR1  (3ZOS) | *ponatinib* | Cc1cc(F)c(C(=O)Nc2ccc(C(=O)N3CCN(C)C(=N)C3O)c(C(F)(F)F)c2)cc1C#Cc1cnc2cc(F)c(F)nn12 | -15.9 | 0.65 | 0.23 | P_Sample1 |
| DDR1  (3ZOS) | *ponatinib* | Cc1cc(O)c(C(=O)Nc2ccc(CN3CC4(F)N=C=C(F)N5C(=N)C3C(F)=C54)c(C(F)(F)F)c2)cc1C#Cc1c(O)nc2cc(F)c(F)nn12 | -15.8 | 0.46 | 0.13 |  |
| DDR1  (3ZOS) | *ponatinib* | Cc1ccc(C(=O)Nc2ccc(C(=O)N3CC4CNC5=NC(C3)N4C5)c(C(F)(F)F)c2)c(F)c1C#Cc1c(O)nc2c(O)c(F)c(F)nn12 | -15.8 | 0.49 | 0.19 |  |
| DDR1  (3ZOS) | *ponatinib* | Cc1cc(F)c(C(=O)Nc2ccc(C(=O)N3CC4NCCN4C(C)(C)C3=O)c(C(F)(F)F)c2)c(F)c1C#Cc1c(O)nc2ccc(F)nn12 | -15.8 | 0.61 | 0.17 |  |
| DDR1  (3ZOS) | *ponatinib* | Cc1c(C#Cc2c(O)nc3cc(F)c(F)nn23)cc(C(=O)Nc2ccc(C(=O)N3CCN(C)C(F)C3)c(C(F)(F)F)c2)c(F)c1F | -15.7 | 0.64 | 0.19 |  |
| DDR1  (3ZOS) | *ponatinib* | Cc1cc(F)c(C(=O)Nc2ccc(C(=O)N3CCN(C)CC3F)c(C(F)(F)F)c2)cc1C#Cc1c(F)nc2cc(F)c(F)nn12 | -15.7 | 0.66 | 0.2 |  |
| DDR1  (3ZOS) | *ponatinib* | Cc1cc(F)c(C(=O)Nc2ccc(C(=O)N3CCN(C)CC3F)c(C(F)(F)F)c2)cc1C#Cc1c(O)nc2c(F)cc(F)nn12 | -15.7 | 0.66 | 0.2 |  |
| DDR1  (3ZOS) | Lead | Cc1cc2c3c(c1)C(C)C(CCNC(=O)c1ccc4[nH]nc(C)c4c1)N3C1=C(C(F)(F)F)NC(=O)CN1C2=O | -12.9 | 0.64 | 0.47 |  |
| DDR1  (3ZOS) | Lead | Cc1n[nH]c2ccc(C(=O)NCCC3C(C)c4cccc5c4N3C3=C(C(F)(F)F)NC(=N)CN3C5=O)cc12 | -12.8 | 0.62 | 0.42 |  |
| DDR1  (3ZOS) | Lead | Cc1n[nH]c2ccc(C(=O)NCCC3C(=O)n4c(c(C(F)(F)F)n(C=O)c(=O)c4=O)N3c3ccccc3F)c(F)c12 | -12.6 | 0.67 | 0.2 | L_sample2 |
| DDR1  (3ZOS) | Lead | Cc1n[nH]c2ccc(C(=O)NCCC3(F)C(=O)N4CC(=O)NC(C(F)(F)F)=C4N3c3ccccc3)c(C)c12 | -12.6 | 0.68 | 0.35 | L_sample1 |
| DDR1  (3ZOS) | Lead | CC1=CC2C(=O)NC(C(F)(F)F)=C3N2C(=O)C1(CCNC(=O)c1cc2oc4n[nH]c2c4c1)N3c1c2cc(c(F)c1C)CN2 | -12.5 | 0.4 | 0.2 |  |
| DDR1  (3ZOS) | Lead | Cc1n[nH]c2ccc(C(=O)NCCC3C(=O)N4CC(=O)NC(C(F)(F)F)=C4N3c3ccccc3)c(C)c12 | -12.5 | 0.7 | 0.49 | L_sample3 |
| DDR1  (3ZOS) | Lead | Cc1n[nH]c2ccc(C(=O)NCCC3(F)C(=O)N4CC(=O)NC(C(F)(F)F)=C4N3c3ccccc3)c(F)c12 | -12.4 | 0.67 | 0.34 |  |
| DDR1  (3ZOS) | Lead | Cc1n[nH]c2ccc(C(=O)NCC(N)C3C(=O)N4CC(=N)NC(C(F)(F)F)=C4N3c3ccccc3)c(C)c12 | -12.4 | 0.63 | 0.35 |  |
| DDR1  (3ZOS) | Lead | Cc1n[nH]c2ccc(C(=O)NCCC3C(C)c4cccc5c4N3C3=C(C(F)(F)F)NC(=O)CN3C5=O)cc12 | -12.4 | 0.65 | 0.49 |  |
| DDR1  (3ZOS) | Lead | CC1c2cccc3c2N(C2=C(C(F)(F)F)NC(=O)CN2C3=O)C1CCNC(=O)c1ccc2[nH]nc(O)c2c1 | -12.4 | 0.64 | 0.41 |  |
| DDR1  (3ZOS) | ZINC12114041 | Cc1cc(F)cc2c1C=CCN2CCN1CC#CC#CC(Nc2nc(C(F)(F)F)c(C)c(=O)[nH]2)CC1 | -13.3 | 0.53 | 0.49 |  |
| DDR1  (3ZOS) | ZINC12114041 | C=C1C(=N)Cc2c1ccc1c2C=CCN1CCN1CCC(Nc2nc(C(F)(F)F)cc(=O)[nH]2)CC1 | -13.2 | 0.7 | 0.6 |  |
| DDR1  (3ZOS) | ZINC12114041 | C=C(C)c1ccc2c(c1)CCCN2CCN1CCC(Nc2nc(C(F)(F)F)c(C)c(=O)[nH]2)CC1F | -13.2 | 0.66 | 0.45 |  |
| DDR1  (3ZOS) | ZINC12114041 | Cc1ccc2c(c1)C1=CCC(NCCN3CCC(Nc4nc(C(F)(F)F)cc(=O)[nH]4)CC3)C1=C2 | -13.1 | 0.69 | 0.6 | V_sample1 |
| DDR1  (3ZOS) | ZINC12114041 | Cc1cccc2c1C=C(F)CN2CCN1CCC(Nc2nc(C(F)(F)F)c(C)c(=O)[nH]2)CC1F | -13.1 | 0.63 | 0.49 | V_sample2 |
| DDR1  (3ZOS) | ZINC12114041 | Cc1cccc2c1C=CCN2CCN1CCC(Nc2nc(C(F)(F)F)c(F)c(=O)[nH]2)CC1F | -13.1 | 0.63 | 0.51 |  |
| DDR1  (3ZOS) | ZINC12114041 | Cc1ccc2c(c1F)C=CCN2CC(=O)N1CCC(Nc2nc(C(F)(F)F)c(C)c(=O)[nH]2)CC1 | -13.1 | 0.75 | 0.66 | V_sample3 |
| DDR1  (3ZOS) | ZINC12114041 | Cc1c(C(F)(F)F)nc(NC2CCN(CCN3CC#CC#CCc4cccc(F)c43)CC2)[nH]c1=O | -13.1 | 0.62 | 0.5 |  |
| DDR1  (3ZOS) | ZINC12114041 | C=C1CC#Cc2ccc3c(c21)C=CCN3CCN1CCC(Nc2nc(C(F)F)cc(=O)[nH]2)CC1 | -13 | 0.65 | 0.64 |  |
| DDR1  (3ZOS) | ZINC12114041 | N=C1Cc2ccc3c(c2C1)C=CCN3CCN1CCC(Nc2nc(C(F)(F)F)cc(=O)[nH]2)CC1 | -13 | 0.71 | 0.62 |  |
| D4DR  (5WIU) | None | C=C1CC(=C=O)C(C2=NN=C3C(N)=C(C(=O)C4=CCC5=C4C(C)CC5=O)CC32)C1=O | -12.7 | 0.5 | 0.57 | N_sample1 |
| D4DR  (5WIU) | None | CC1CCC(=O)C(CC2=C(C(C)C3CC4=C=CCC(C)C4=CC3=O)CCCC2=O)C1 | -11.4 | 0.52 | 0.56 |  |
| D4DR  (5WIU) | None | C=C(C)CC1CC(C(=O)C2=CC=C(C3C=CC4=C3CC3=C4CNC3)C2)C(O)C1=O | -11.3 | 0.51 | 0.71 |  |
| D4DR  (5WIU) | None | CC1=C(C)C2CC(=O)C(C(=O)CC3CC=C4CC(=O)CC=C43)=CC2=C1 | -11.3 | 0.58 | 0.73 |  |
| D4DR  (5WIU) | None | CC1=C2C(=O)C(=O)C(C(=O)CC3=CC4=C(CC=N4)C3=N)=C=C2C=C(CCC(C)C)C1 | -11.3 | 0.61 | 0.41 | N_sample2 |
| D4DR  (5WIU) | None | C=C1C(=N)C(C)=C(CC2=NC(C3=C4CCCC=C4CC(=C)C3N)=CC2=O)C1=O | -11.2 | 0.57 | 0.59 |  |
| D4DR  (5WIU) | None | N=C1CC(C=CC(=O)CCC2=C3CCCC=C3C#CC2=O)=NNC1=O | -11.1 | 0.6 | 0.45 |  |
| D4DR  (5WIU) | None | CC1=C(C2=CCC(=C3CC(=O)C(C)=C3C3C=CCC3=O)CC2)CCCC1 | -11 | 0.64 | 0.6 |  |
| D4DR  (5WIU) | None | CC(=O)C1=C(CC2CNC(=O)CC2=O)C(=O)C(=N)C2=C1CC#CC2=O | -10.9 | 0.56 | 0.32 |  |
| D4DR  (5WIU) | None | CC1CC=C(C2=CC=CC3C(C=C2)CC2C(=O)CC(C)C23)CC1 | -10.8 | 0.58 | 0.65 |  |
| D4DR  (5WIU) | ZINC12203131 | N#CCc1c(C=C=O)cc2c(=O)c3c4oc2c1N(C(N)c1noc(-c2c5cc(c(F)c2F)C=C5)n1)C4OO3 | -14.8 | 0.42 | 0.27 |  |
| D4DR  (5WIU) | ZINC12203131 | CCc1ccc2c(c1F)-c1nc(no1)C2(F)N(Cc1cc(=O)c2c(C)c3c(cc2o1)C(=N)CCC3(F)F)N(F)F | -14.8 | 0.51 | 0.14 |  |
| D4DR  (5WIU) | ZINC12203131 | Cc1cc(N)c(C)c2cc3c(=O)c4c(oc3cc12)C(=O)N=C4c1noc(-c2c(O)cccc2F)n1 | -14.3 | 0.74 | 0.29 | Z_sample1 |
| D4DR  (5WIU) | ZINC12203131 | O=Cc1cc2c(c3c1C1=CC3=C=N1)OC1=C(C2)C(c2noc(-c3ccc(F)c(F)c3)n2)=NC1=O | -14.2 | 0.58 | 0.56 |  |
| D4DR  (5WIU) | ZINC12203131 | Cc1cc2c(cc1C)OC1=C(C2)C(c2noc(-c3ccc(F)c4cn[nH]c34)n2)=NC1=O | -13.8 | 0.73 | 0.54 | Z_sample2 |
| D4DR  (5WIU) | ZINC12203131 | C=C1C2=C(Oc3cc(C)c(C4(F)C=CC=NN4OF)cc31)C(=O)N=C2c1noc(-c2ccc(F)cc2C)n1 | -13.7 | 0.61 | 0.45 |  |
| D4DR  (5WIU) | ZINC12203131 | Cc1cc2c(c(F)c1C)OC1=C(C2)C(c2noc(-c3ccc(F)c(F)c3C)n2)=NC1=O | -13.7 | 0.73 | 0.61 |  |
| D4DR  (5WIU) | ZINC12203131 | Cc1cc2c(cc1C)OC1=C(C2)C(c2noc(-c3cccc4c3C=CC4)n2)=NC1=O | -13.7 | 0.74 | 0.65 |  |
| D4DR  (5WIU) | ZINC12203131 | Cc1cc2c(c3c1C=CC3)OC1=C(C2)C(c2noc(-c3cccc(F)c3)n2)=NC1=O | -13.7 | 0.72 | 0.65 |  |
| D4DR  (5WIU) | ZINC12203131 | Cc1cc2c(cc1C)OC1=C(C2)C(c2noc(-c3ccc(F)c4c3C#CC4)n2)=NC1=O | -13.7 | 0.67 | 0.6 |  |
| PARP-1  (4R6E) | E7449 fragment | Cc1cc(C)c2c(=O)[nH]nc3[nH]c(CC4=C5CC6=C4C(=O)CC(=C6)C5=O)nc1c32 | -15.1 | 0.58 | 0.71 |  |
| PARP-1  (4R6E) | E7449 fragment | Cc1c2c(c3nc(C4=CC(=N)C(=O)C(=O)C4)[nH]c4n[nH]c(=O)c1c43)CN(F)N=C2NF | -15 | 0.61 | 0.33 |  |
| PARP-1  (4R6E) | E7449 fragment | Cc1c(C)c2nc(C3CC(=O)C(C(=O)C4C=C5C(=N)C=C4C5=O)C3)[nH]c3n[nH]c(=O)c(c1C)c32 | -14.9 | 0.48 | 0.52 |  |
| PARP-1  (4R6E) | E7449 fragment | Cc1cc(C)c2c(=O)[nH]nc3[nH]c(C4CC(=O)C5=C6C(=O)C(=N)C5=CC64)nc1c32 | -14.8 | 0.49 | 0.59 |  |
| PARP-1  (4R6E) | E7449 fragment | C=C1C=C2C(=N)C(c3nc4c(C)c(C)c(C)c5c(=O)[nH]nc([nH]3)c45)C1CC2=O | -14.7 | 0.52 | 0.61 | A_sample1 |
| PARP-1  (4R6E) | E7449 fragment | Cc1c(C)c2nc(C3CC(=N)C4=CC(F)C=C3C4=O)[nH]c3n[nH]c(=O)c(c1C)c32 | -14.6 | 0.5 | 0.59 |  |
| PARP-1  (4R6E) | E7449 fragment | CC1=C(c2nc3c(C)c(C)c(C)c4c(=O)[nH]nc([nH]2)c34)CC2(C#CC(=O)C2=N)C1 | -14.6 | 0.52 | 0.44 |  |
| PARP-1  (4R6E) | E7449 fragment | Cc1c(C)c2nc(C3=C4CC5(C=C(C3)C4=O)CCC(=O)C5)[nH]c3n[nH]c(=O)c(c1C)c32 | -14.6 | 0.5 | 0.63 |  |
| PARP-1  (4R6E) | E7449 fragment | Cc1c(C)c2nc(C3CC4=C(CCC(=O)C4=O)N3)[nH]c3n[nH]c(=O)c(c1C)c32 | -14.6 | 0.65 | 0.56 |  |
| PARP-1  (4R6E) | E7449 fragment | Cc1c(C)c2nc(C3CC4=C(CCC(=N)C4=O)N3)[nH]c3n[nH]c(=O)c(c1C)c32 | -14.6 | 0.62 | 0.52 |  |

*Lead: SCHEMBL18373876

Supplementary Table 2. Top 10 compounds of the virtual screening result against DDR1 (top) and D4 dopamine receptor (bottom).

| Compound | Energy | nRot | isLeadLike | HBA | HBD | LogP | MW | TPSA |
| --- | --- | --- | --- | --- | --- | --- | --- | --- |
| ZINC12114041 | -12.1 | 5 | Y | 7 | 2 | 2.07 | 435.4428 | 81.33 |
| ZINC8860262 | -11.6 | 5 | Y | 7 | 1 | 2.36 | 423.5113 | 146.28 |
| Pubchem SID:47199487 | -11.1 | 2 | Y | 4 | 0 | 3.64 | 273.2887 | 51.81 |
| ZINC2917344 | -10.7 | 5 | Y | 7 | 1 | 2.53 | 390.4351 | 77.63 |
| ZINC2661770 | -10.7 | 7 | Y | 6 | 1 | 3.48 | 381.4002 | 73.22 |
| ZINC7424951 | -10.5 | 3 | Y | 5 | 1 | 3.55 | 306.2938 | 67.6 |
| Pubchem SID:24800280 | -10.4 | 7 | Y | 6 | 1 | 2.71 | 350.4143 | 80.91 |
| Pubchem SID: 24820230 | -10.3 | 5 | Y | 5 | 1 | 3.26 | 319.3572 | 60.06 |
| Pubchem SID: 17386916 | -10.3 | 7 | Y | 6 | 0 | 3.65 | 420.4809 | 104.81 |

| Compound | Energy | nRot | isLeadLike | HBA | HBD | LogP | MW | TPSA |
| --- | --- | --- | --- | --- | --- | --- | --- | --- |
| Pubchem SID: 24323848 | -12.5 | 2 | Y | 5 | 2 | 3.49 | 407.5086 | 126.7 |
| ZINC794507 | -12 | 5 | Y | 7 | 1 | 3.22 | 426.4672 | 84.3 |
| Pubchem SID: 24370346 | -12 | 6 | Y | 6 | 2 | 3.72 | 413.4684 | 83.96 |
| ZINC12203131 | -11.9 | 4 | Y | 7 | 1 | 3.17 | 375.3774 | 98.23 |
| Pubchem SID: 49736041 | -11.9 | 4 | Y | 7 | 1 | 2.67 | 408.4255 | 78.53 |
| ZINC12202071 | -11.9 | 3 | Y | 6 | 1 | 3.5 | 377.4164 | 113.33 |
| ZINC8619126 | -11.9 | 6 | Y | 6 | 1 | 3.85 | 429.443 | 81.06 |
| ZINC6660994 | -11.9 | 4 | Y | 6 | 0 | 3.76 | 424.4647 | 62.99 |
| Pubchem SID: 24782939 | -11.8 | 3 | Y | 5 | 0 | 3.93 | 368.4311 | 50.5 |
| Pubchem SID: 26649638 | -11.8 | 3 | Y | 6 | 2 | 2.08 | 377.4827 | 69.22 |

Supplementary Fig. 1 Binding interactions of ZINC12114041 and V_sample1 with the PDB structure 3ZOS.


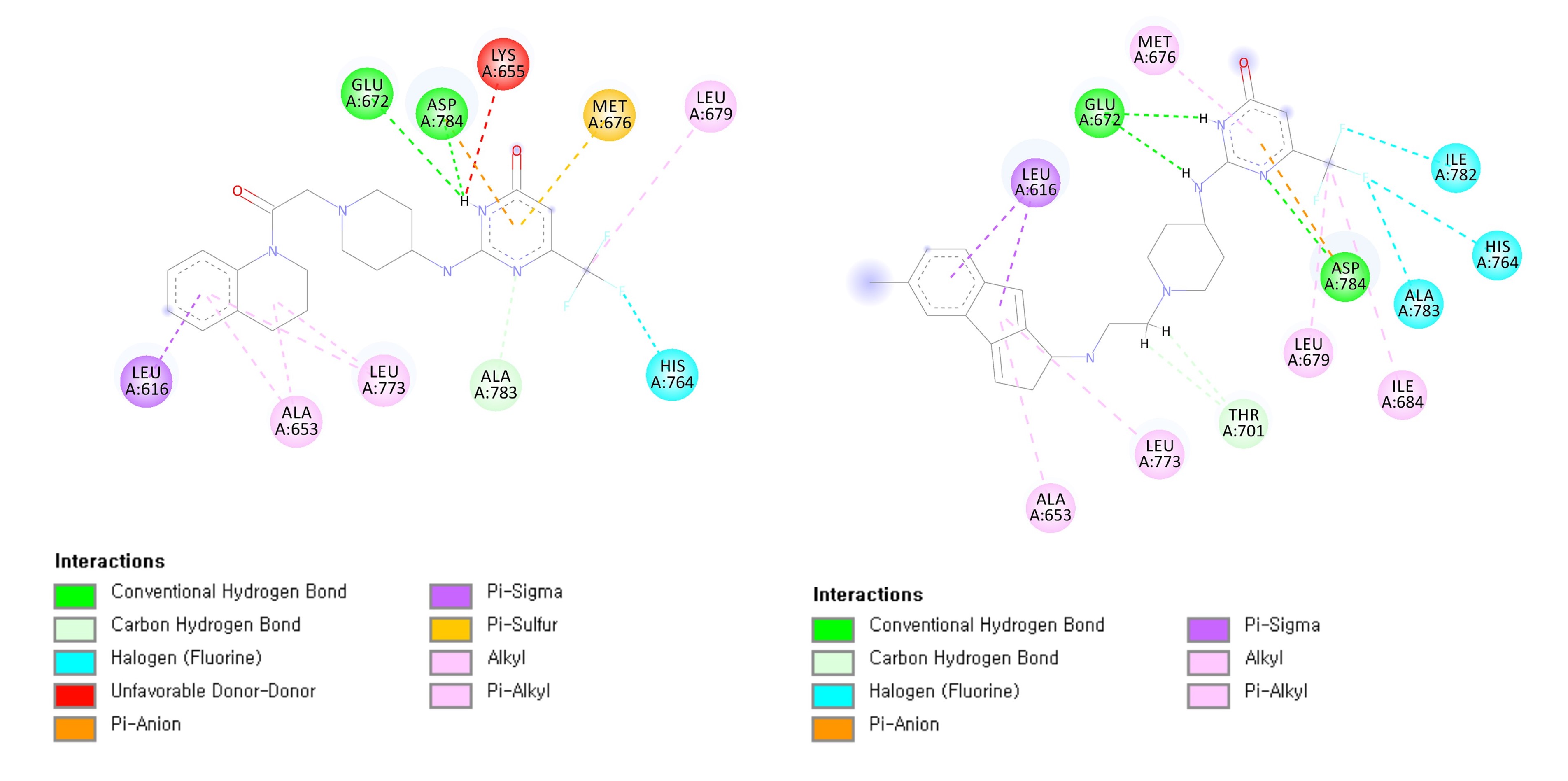


Supplementary Fig. 2 The optimized result of the fragment of e7449. (Left) a sample from optimized results (A_sample1) with QuickVina 2 score -14.6 kcal/mol, synthetic accessibility (SA) score 0.65, and quantitative estimate of drug-likeness (QED) score 0.56. Binding pose of A_sample1 docked to the structure 4R6E (PARP-1). (Right) Binding interactions of A_sample1 to 4R6E.


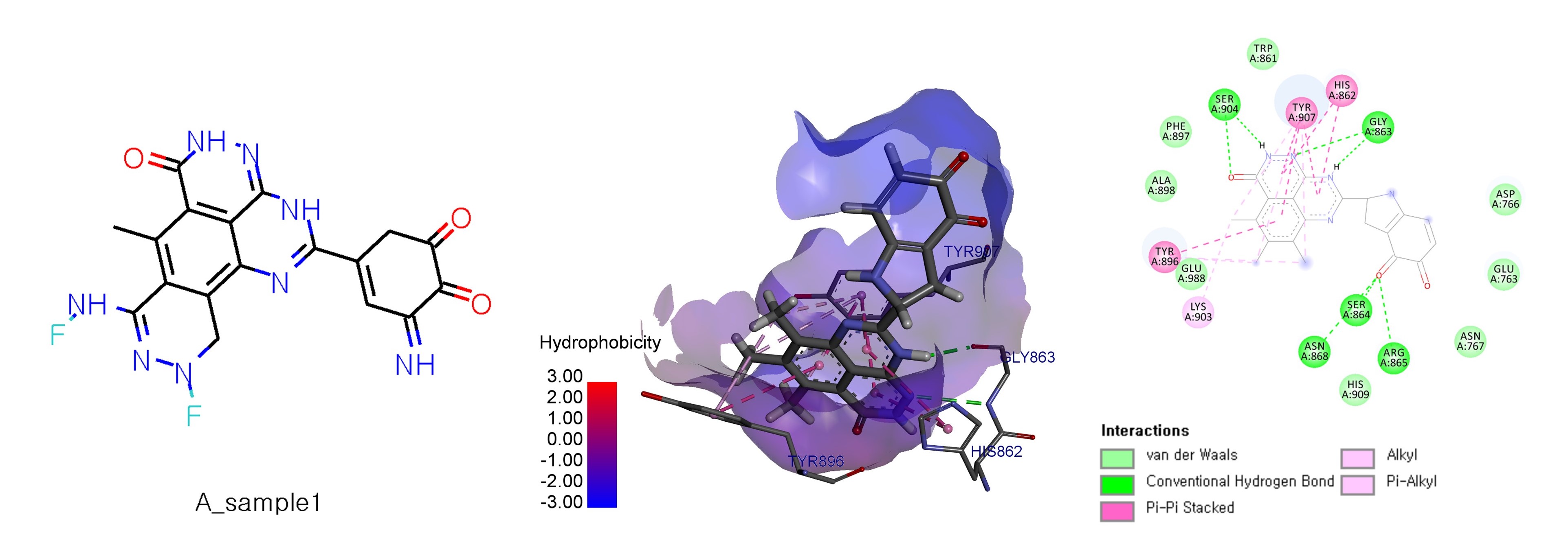


Supplementary Data 1. Hyperparameters of MolDQN used in MORLD.

{
 "allow_removal": true,
 "allow_no_modification": true,
 "allow_bonds_between_rings": false,
 "allowed_ring_sizes": [5, 6],
 "replay_buffer_size": 5000,
 "learning_rate": 0.01,
 "learning_rate_decay_steps": 10000,
 "learning_rate_decay_rate": 0.9,
 "batch_size": 32,
 "learning_frequency": 4,
 "update_frequency": 50,
 "grad_clipping": 10,
 "gamma": 1.0,
 "discount_factor": 0.95,
 "double_q": true,
 "num_bootstrap_heads": 8,
 "prioritized": true,
 "prioritized_alpha": 0.6,
 "prioritized_beta": 0.4,
 "prioritized_epsilon": 0.000001,
 "fingerprint_radius": 3,
 "fingerprint_length": 2048,
 "dense_layers": [1024, 512, 128, 64],
 "activation": "relu",
 "optimizer": "Adam",
 "batch_norm": false,
 "save_frequency": 200,
 "max_num_checkpoints": 10
}
