# Supplementary figures and images for: Autonomous molecule generation using reinforcement learning and docking to develop potential novel inhibitors
Source: Sci Rep. 2020 Dec 16;10:22104. doi: 10.1038/s41598-020-78537-2 (PMC7744578; doi:10.1038/s41598-020-78537-2)

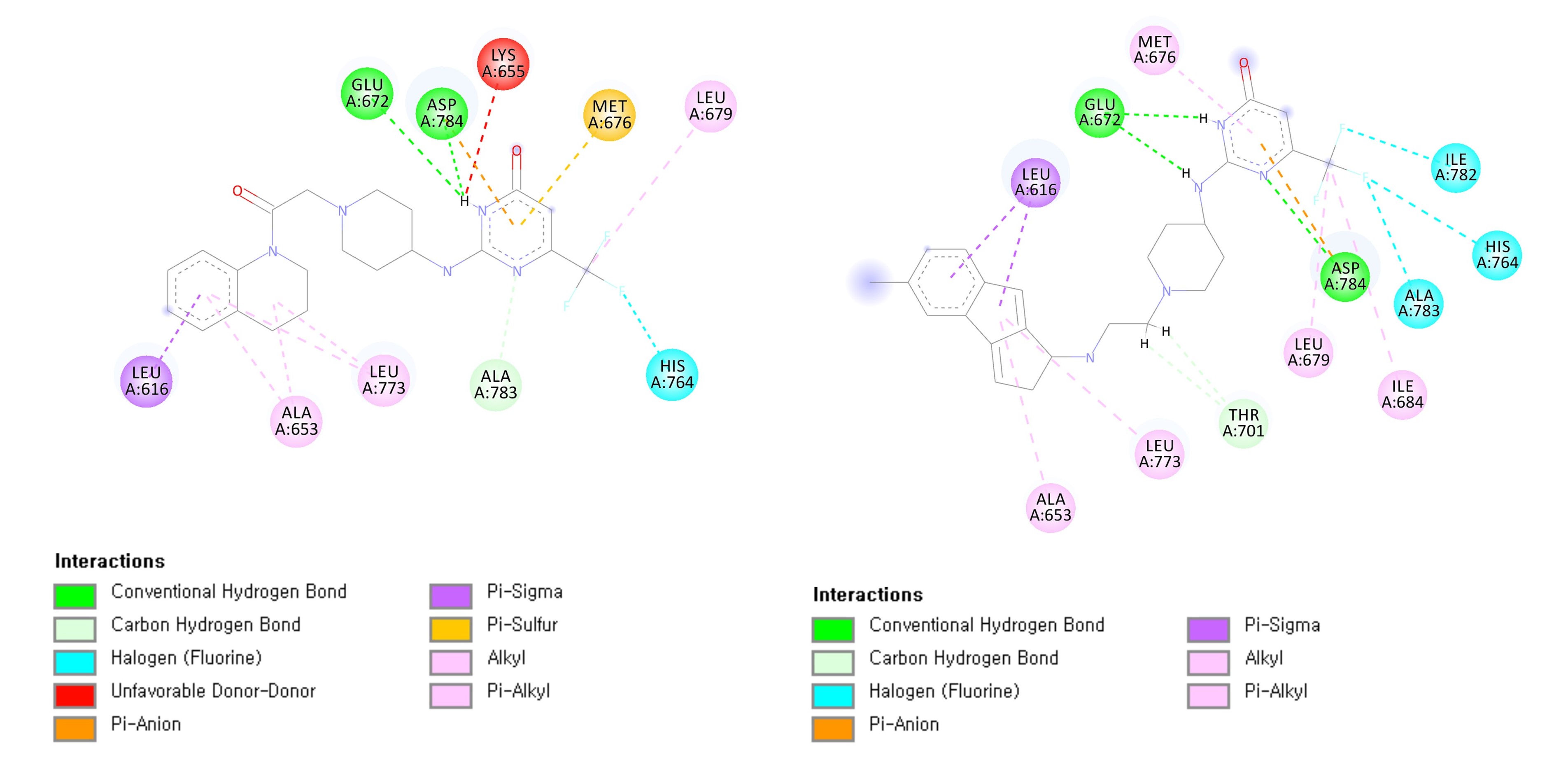

Supplement: Supplementary file 2 — Supplementary Figure S1. [file 41598_2020_78537_MOESM2_ESM.jpg]

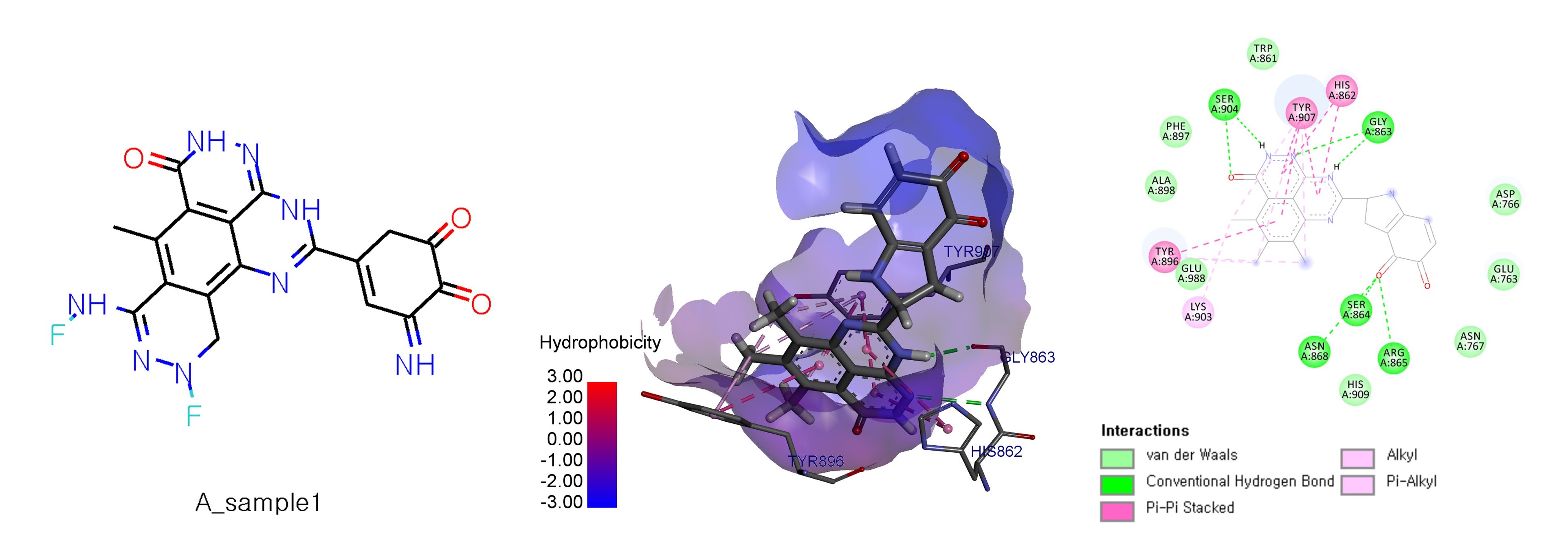

Supplement: Supplementary file 3 — Supplementary Figure S2. [file 41598_2020_78537_MOESM3_ESM.jpg]
